# Gene Expression Regulation and the Signal Transduction of Programmed Cell Death

**DOI:** 10.3390/cimb46090612

**Published:** 2024-09-16

**Authors:** Saqirile, Yuxin Deng, Kexin Li, Wenxin Yan, Ke Li, Changshan Wang

**Affiliations:** Laboratory of Reproductive Regulation & Breeding of Grassland Livestock, School of Life Science, Inner Mongolia University, 49 Xilingol South Road, Yu Quan District, Hohhot 010020, China; sqrl1009@163.com (S.); dyx20240322@163.com (Y.D.); kexinli@mail.imu.edu.cn (K.L.); 15065525077@163.com (W.Y.); like990614@163.com (K.L.)

**Keywords:** programmed cell death, gene expression regulation, signal transduction, cancer

## Abstract

Cell death is of great significance in maintaining tissue homeostasis and bodily functions. With considerable research coming to the fore, it has been found that programmed cell death presents in multiple modalities in the body, which is not only limited to apoptosis, but also can be divided into autophagy, pyroptosis, ferroptosis, mitotic catastrophe, entosis, netosis, and other ways. Different forms of programmed cell death have disparate or analogous characteristics with each other, and their occurrence is accompanied by multiple signal transduction and the role of a myriad of regulatory factors. In recent years, scholars across the world have carried out considerable in-depth research on programmed cell death, and new forms of cell death are being discovered continually. Concomitantly, the mechanisms of intricate signaling pathways and regulators have been discovered. More critically, cancer cells tend to choose distinct ways to evade cell death, and different tumors adapt to different manners of death. Therefore, targeting the cell death network has been regarded as an effective tumor treatment strategy for a long time. The objective of our paper is to review the signaling pathways and gene regulation in several typical types of programmed cell death and their correlation with cancer.

## 1. Introduction

Cell death plays a pivotal role in physiological development, maintenance of homeostasis, establishment and maintenance of immune tolerance state, tumor monitoring, and pathological processes. It can be divided into programmed cell death and non-programmed cell death, such as cell necrosis. According to the definition and classification by morphological standards recommended by the Nomenclature Committee on Cell Death (NCCD) in 2009 [1], the typical modalities include apoptosis, autophagy, cornification, pyroptosis, ferroptosis, etc. The atypical forms are extrinsic apoptosis, intrinsic apoptosis, anoikis, mitotic apoptosis, entosis, netosis, parthanatos, and necroptosis, etc. Programmed cell death occurs in a variety of ways, each with different morphological and physiological characteristics, but all of them are orchestrated by signaling pathways and regulators, and anomalous changes in key links will lead to pathological processes, even cancer.

## 2. Overview of Programmed Cell Death

### 2.1. History of Programmed Cell Death Research

Programmed cell death is commonly found in the development process of various organisms. It is an active and orderly way of cell death that is genetically determined to maintain a stable intracellular environment. It was originally proposed by developmental biologists as a way of cell death [2]. This physiological and selective cell death occurs through a series of gene activation, expression, and regulation processes. That is, when cells are stimulated by internal or external environmental factors, “suicide” protection is initiated through gene regulation to remove unnecessary cells or cells that are about to undergo specialization in the body.

As early as 1972, J. F. R. Err, A. H. Wollie, and A. R. Culrie formally coined the term apoptosis and emphasized that this pattern of cell death is a normal physiological process [3]. Subsequently, various forms of programmed cell death have been continuously discovered, and the molecular mechanisms of different modalities have gradually become clear, providing ideas for the treatment of some tumors and other diseases and drug targets in the treatment.

### 2.2. Types of Programmed Cell Death

Programmed cell death can be induced by physical factors, such as radiation or temperature stimulation, and by reactive oxygen groups or by chemical and biological factors, such as tumor necrosis factor. Cells stimulated by apoptotic inducers undergo different apoptotic pathways. The protease caspase (cysteine aspartic acid-specific protease) family plays an indispensable role in this process. They constitute the cascade molecular network of intracellular apoptotic signals, selectively cut regulatory proteins or structural proteins, activate or inactivate the target proteins, and enable the cells to enter the execution stage of apoptosis [4].

Based on this, programmed cell death can be divided into two types: caspase-dependent apoptosis and caspase-independent apoptosis [5]. Caspase-dependent cell apoptosis is the classic apoptosis, mainly including apoptosis, pyroptosis, and cornification; caspase-independent apoptosis mainly includes autophagy, ferroptosis, mitotic catastrophe, entosis, netosis, parthanatos, and necroptosis, etc. [6]. And we summarize the characteristics and differences about different modalities of cell death in Table 1.

## 3. Apoptosis

### 3.1. Signal Transduction of Apoptosis

Cell apoptosis is of paramount significance in cell death and is the pillar regulator of tissue homeostasis and physiological development. Apoptosis is orchestrated by a number of genes and signaling pathways, whose dysfunction results in pathological processes, including cancer. Studies on the molecular mechanism of apoptosis have shown that apoptosis can be divided into caspase-dependent apoptosis and caspase-independent apoptosis.

#### 3.1.1. Caspase-Dependent Apoptosis

Caspase-dependent apoptosis can be divided into exogenous pathways initiated by death receptors and endogenous pathways initiated by mitochondria. In exogenous pathway, death ligands are mainly members of the tumor necrosis factor (TNF) family [7], while death receptors have multiple members. At present, the main eight types of death receptors include TNF-R1, Fas (APO-1/CD95), DR3 (APO-3, WSL-1, TRAMP), TRAIL-R1, DR-5, DR-6, and EDR-R (ectodermal dysplasia receptor) [8]. When the death receptors on the cell membrane recognize the related ligands, their cytoplasmic death domains bind to ligands and facilitate trimerization to induce apoptosis and inflammation.

Fas is a representative member of the death receptor family. After binding to its ligand FasL, polymerization takes place. After polymerization, pro-caspase-8 is recruited to the cell membrane through the adaptor protein FADD to form the death-inducing signal complex (DISC). Caspase-8 is activated in this complex by autoactivation and then cutting downstream effectors caspases for a combination reaction, that is, cutting downstream pro-caspase-3 to make it active and then the activated caspase-3 acts on a series of downstream target proteins to regulate the occurrence of apoptotic events. At the same time, the activated caspase-8 also cuts through the signaling molecules Bid to transmit apoptosis signals to mitochondria to trigger endogenous apoptosis pathways.

**Figure 1:**
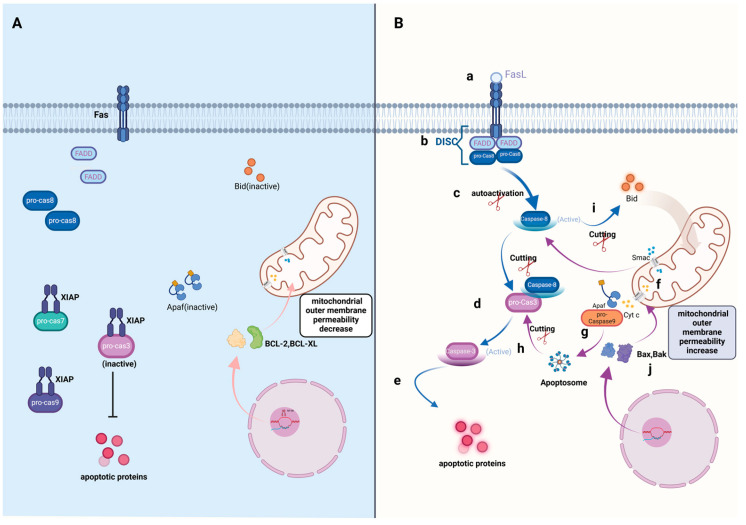
Apoptotic pathways (created with BioRender.com).

As shown in Figure 1, in the endogenous pathway, the change in mitochondrial outer membrane permeability is decisive. When cells perceive devastating signals, like DNA damage, oxidants, ceramides, etc., changes in membrane permeability lead to the release of apoptotic factors, and cytochrome C (Cyt C) is the major “helmsman” of intrinsic apoptosis [9]. After Cyt C is released, it will combine with another apoptotic factor, Apaf-1. Apaf-1 recruits pro-caspase-9 to form an apoptosome through its structural domain CARD. The pro-caspase-9 is activated by self-cleavage in the apoptosome, followed by the further cleavage of downstream pro-caspase-3/7, causing apoptosis.

**During the apoptotic process, as shown in part B**, (a) the death ligand FasL from the extracellular environment binds to the death receptor Fas, which is located in the cell membrane and triggers the exogenous apoptotic pathway (the pathway that is presented by blue arrows). (b) Then, Fas polymerizes and recruits the adaptor protein FADD and pro-caspase-8 to form the death-inducing signaling complex (DISC). (c) The formation of DISC facilitates pro-caspase-8 to self-cut and activate (caspase-8). (d) The activated caspase-8 cuts the downstream effector pro-caspase-3 to make it active (caspase-3). (e) Finally, the activated caspase-3 acts on a series of downstream target proteins to regulate the occurrence of apoptotic events. The endogenous pathway (represented by purple arrows) starts with mitochondria when cells receive devastating signals. (f) Changes in mitochondrial membrane permeability lead to the release of apoptotic factor cytochrome C (Cyt C). (g) Cyt C combines with the apoptotic factor Apaf-1 and pro-caspase-9 to form apoptosome. (h) The pro-caspase-9 is activated in the apoptosome, followed by the further cleavage of downstream pro-caspase-3, causing apoptosis. (i) In addition, Bid could be cut and activated by caspase-8 to an active state and facilitate the release of Cyt C. (j) The pro-apoptotic proteins Bak and Bax could interact with the mitochondrial membrane protein VDAC and promote the release of Cyt C.

**In normal cells without apoptosis, as shown in part A**, the exogenous and endogenous apoptotic pathways are inactive. The anti-apoptotic proteins BCL-2 and BCL-XL inhibit the activity of pro-apoptotic proteins, thereby inhibiting the opening of mitochondrial membrane channels.

#### 3.1.2. Caspase-Independent Apoptosis

Studies have shown that mammalian cells can still undergo apoptosis even after caspases are knocked out or inhibited, suggesting that caspase-independent apoptosis still exists in the cells. Current studies have found that apoptotic induction factor (AIF) plays a key role in the non-dependent cell death of caspases [10], and it is the first cloned protein that can induce the apoptosis of caspase-independent cells. Mitochondria release these apoptotic factors directly into the nucleus, causing DNA fragmentation. At the same time, granzyme A causes the SET complex located in the endoplasmic reticulum to dissociate, the latter transfers to the nucleus, and then is activated to cut DNA.

Caspase-independent apoptosis leads to DNA fragmentation with a size of about 5 × 10^4^ bp, which is different from the 200 bp fragment of classical apoptosis [11]. However, both caspase-dependent and caspase-independent apoptosis result in cell apoptosis and thus elimination.

The apoptosis pathways in cells are correlated with each other. As shown in Figure 1, in the caspase-dependent apoptosis pathway, when caspase-8 is activated, it can cut the pro-apoptotic factor Bid to activate it, and then enter the mitochondria to promote the release of apoptotic-related factors to generate the cascade amplification effect of apoptosis. At the same time, when the endogenous apoptotic pathway is activated, mitochondria release a pro-apoptotic factor called Smac, which activates the pro-caspase-8 enzyme to carry out the exogenous apoptotic pathway. As for the entire apoptosis system, mitochondria play a key role in both caspase-dependent and non-dependent apoptosis pathways.

### 3.2. Gene Regulation of Apoptosis

#### 3.2.1. BCL-2 Family

The B-cell lymphoma gene 2 (BCL-2) protein family is one of the most valued oncogenes in cell apoptosis research, among which there are both genes that can inhibit apoptosis or promote apoptosis, and all the members contain one or more BH (Bcl-2 Homology) domains [12]. According to the structure and function, BCL-2 family can be divided into three subfamilies: Bcl-2 subfamily, Bax subfamily, and BH3 subfamily. Among them, the BCL-2 subfamily plays an inhibitory role in cell apoptosis, and most of them have four BH structural domains (BH1-BH4) [13]. The other two subfamilies play a role in promoting apoptosis, and the BH domain, found to markedly be different, has something in common with the BCL-2 subfamily: the absence of the BH4 domain. For example, Bax and Bak of the Bax subfamily all have three BH domains, including BH1-BH3. BH3 subfamily members such as Bad, Bid, Noxa, etc., only have the BH3 domain. These phenomena indicate that the BH4 domain plays an important part in anti-apoptosis.

The oligomerization pro-apoptotic factors Bak and Bax in the BCL-2 family can regulate the permeability of the mitochondrial outer membrane and release Ca^+^ at the same time, so that Cyt C can be released into the cytoplasm and induce the caspase cascade effect leading to cell apoptosis. The mechanism may be that Bak and Bax transfer to the mitochondrial membrane and interact with the voltage-dependent anion channel (VDAC) on the membrane to open the channel and release Cyt C [14]. As homologous proteins of Bax and Bad, BCL-2, which can inhibit cell apoptosis, can combine with each other to form heterodimers and play a role of mutual inhibition. Therefore, the ratio between Bax, Bad, and BCL-2 also determines whether the cell moves on to apoptosis or survival.

#### 3.2.2. NF-kB Signaling

Nuclear factor κB (NF-κB) is a transcription factor widely expressed in mammalian cells and is classified as part of the NF-κB/Rel protein family [15]. It is closely related to cell apoptosis, which functions as a double-edged sword in cell apoptosis.

The NF-κB coding gene has traditionally been considered to inhibit apoptosis and is central to a number of anti-apoptotic and pro-survival signaling pathways. It plays a direct role by activating the transcription or expression of anti-apoptotic target genes or an indirect role by inhibiting the transcription or expression of pro-apoptotic target genes. NF-κB binds to its inhibitor I-κB (inhibitor κB) and is located in the cytoplasm in an inactive state. When the cell receives external signals, NF-κB is activated to initiate gene transcription within the nucleus and inhibit cell apoptosis. Different from this, NF-κB binds to coding genes of BCL-2 and BCL-XL. The activated NF-κB increases the expression of BCL-2 and BCL-XL in mitochondria, leading to decreased mitochondrial membrane permeability, blocking the release of Cyt C and thereby inhibiting cell apoptosis [16]. The X-linked inhibitor of apoptosis protein (XIAP), a key factor of NF-κB, specifically binds to and inhibits the activation of pro-caspase-3/7/9, thereby blocking the release of Cyt C and inhibiting cell apoptosis [16]. The Fas/FasL signaling pathway is regulated by NF-κB at the transcriptional level. When the recruitment of NF-κB to the Fas region is reduced, the Fas/FasL gene is expressed in large amounts to induce apoptosis. Therefore, NF-κB inhibits the apoptosis induced by the Fas/FasL signaling pathway. In the endoplasmic reticulum stress pathway, NF-κB can inhibit apoptosis by activating and down-regulating intracellular reactive oxygen species (ROS) levels [17], thereby inhibiting the JNK cascade response.

Further studies of NF-κB and apoptosis have shown that NF-κB can promote apoptosis under certain conditions. The first way is to directly activate the pro-apoptotic molecules in cells. It was found that the overexpression of IKK in T cells leads to the activation of NF-κB, which induces the expression of TNF-related apoptosis-inducing ligand (TRAIL) [18], initiating the apoptosis pathway. The second approach is to inhibit the expression of anti-apoptotic molecules. NF-κB also inhibits the anti-apoptotic proteins such as XIAP when stimulated by ultraviolet light or drugs, thus reducing their anti-apoptotic effects. The last way is through NF-κB, which can activate tumor suppressors (cylindromatosis, CYLD), promoting its activation and expression. CYLD, on the other hand, degrades the polyubiquitinated chain of K63 on TRAF2/6 and IKK when it acts as a deubiquitinating enzyme to lead to the breakdown of the IKK complex, and finally inhibits the activation of NF-κB as a negative feedback, thus providing an apoptotic environment [19].

#### 3.2.3. p53

The transcription factor p53 is an important tumor suppressor gene and apoptotic factor, which can block the cell cycle, promote cell apoptosis, and maintain genomic stability [20]. Apoptosis signals obtained by mitochondria often come from intracellular p53, which can activate the transcription of positive apoptotic regulators, such as Bax, Apaf-1, and caspase-independent apoptosis factor PIG3 (p53-inducible gene 3), or inhibit the transcription of anti-apoptotic factors, such as BCL-2 and Survivin [21]. These proteins’ duty is to regulate apoptosis.

The activity of p53 in cells is often low. When cells are stimulated by ultraviolet rays or their DNA is accidentally damaged, it will be considerably activated, blocking the cell cycle and even causing cell apoptosis. p53 can up-regulate the expression level of Bax and down-regulate the expression of BCL-2 to promote apoptosis.

#### 3.2.4. Other Regulatory Factors

Numerous important apoptosis-related molecules also exist in cells. For example, Ced9 in nematodes can inhibit apoptosis by inhibiting Cyt C release in mitochondria [22]. cIAP family in the cells of drosophila can directly interact with caspase to block its substrates’ cutting ability [23]. In addition, the caspase inhibitor v-FLIPs, evolved in viruses, can interact with the adaptor protein FADD, making caspase-8 unable to come into contact with FADD and inhibiting exogenous apoptosis [24].

### 3.3. Implications of Apoptosis in Cancer

Due to the considerable effects and functions of apoptosis in cell fate, scientists regard this process as an important breakthrough in cancer treatment and have developed a large number of targeted drugs. A study based on a ginseng saponin derivative found that it showed anti-tumor activity in prostate cancer cells by inducing the cell apoptosis of the BCL-2 family-mediated mitochondria pathway. And the result indicated that natural products obtained from medicinal herbs have great potential in cancer treatment, with fewer side effects [25]. Acute myeloid leukemia (AML) is an aggressive hematopoietic malignancy, and TRAIL is regarded as a potential anticancer drug but has limited effectiveness. Scientists found that the simultaneously employment of TRAIL and the BCL-2 inhibitor venetoclax (VEN) could present a strong synergistic antileukemic activity in AML cells by stimulating extrinsic and intrinsic apoptosis signaling pathways [26]. In breast cancer, PRC2-induced trimethylating histone H3 at the lysine 27 residue (H3K27me3) leads to the transcriptional repression of GATA4 accompanied by FAS inactivation; this interplay helps cancer cells to resist FasL-induced apoptosis [27]. Targeting apoptosis pathway is, nonetheless, a good way to overcome cancer; however, scientists have found that cancer cells present different sensitivities to related drugs. With the discovery of new targeted small molecules and the exploitation of natural drugs, experiments have shown that combination drug therapy show a better efficacy in tumor treatment.

## 4. Autophagy

### 4.1. Signal Transduction of Autophagy

Cell autophagy refers to the cells’ sensing of external stimulation, like starvation and nutrition deprivation, and then their triggering of self-degradation by the fusion of lysosomes with autophagic vesicles (termed autophagosomes). Then, the autophagosomes decompose excessive cellular contents into small molecules through hydrolytic enzyme digestion to be able to be recycled. The autophagy pathway is often selected for the degradation of protein aggregates or abnormal organelles, which can be mainly divided into: micro-autophagy, chaperone-mediated autophagy (CMA), and macro-autophagy, with autophagy being primarily divided into xenophagy and mitophagy [28]. Among them, macro-autophagy is the most common typical autophagy process. Micro-autophagy refers to the lysosome’s active and direct invagination of the lysosomal membrane, thus engulfing its targets. Chaperon-mediated autophagy requires the involvement of chaperones, such as Hsp70, to assist the entry of unfolded proteins into lysosomes for protein clearance. Based on the study of the molecular mechanism of autophagy, it can be divided into two categories: the mTOR-dependent autophagy pathway and other pathways independent of mTOR.

#### 4.1.1. Upstream Pathway of Autophagy

Rapamycin-sensitive mTOR plays an important role in autophagy [29]. Since the autophagy pathway mediated by mTOR was discovered, scientists have turned their attention to the factors that can fine-tune mTOR, namely the upstream pathway of mTOR. The upstream pathway of mTOR-dependent autophagy can be divided into the PI3K-Akt-mTOR pathway and AMPK-TSC1/2 mTOR pathway [30]. Both of these signaling pathways inhibit mTOR signaling to activate autophagy or activate mTOR signaling to inhibit autophagy under the action of protein kinases or signaling molecules.

Studies have found that there are also many autophagy pathways that are not dependent on mTOR. For example, PI3K class I, a negative regulator of autophagy, can directly participate in the regulation of autophagy, or the participation of Beclin-1 combined with the UV-RAG complex of VPS34 Class 3 PI3K can regulate autophagy [31].

#### 4.1.2. Autophagy Signal Transduction Pathway

**Figure 2:**
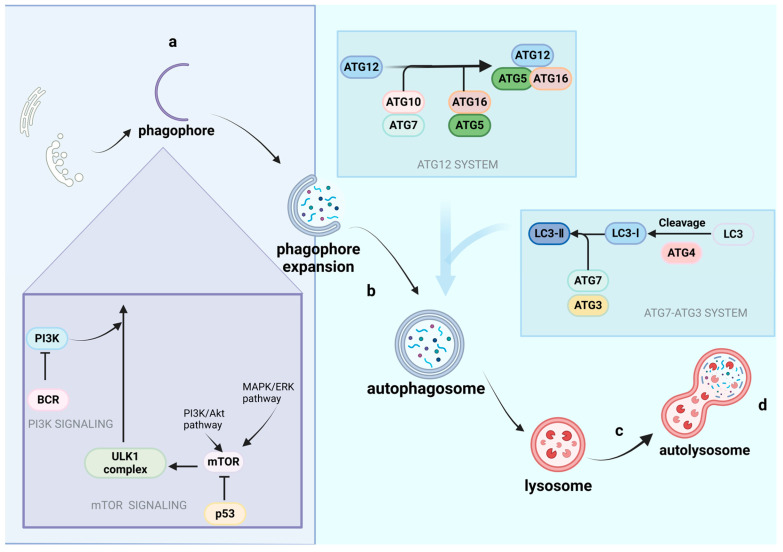
Autophagic pathways (created with BioRender.com).

As shown in Figure 2, the classical process of autophagy mainly includes the formation and extension of phagophores, membrane nucleation, autophagosome maturation, fusion with the lysosome, and the degradation of cargo by lysosomal acid proteases.

Under nutritionally adequate conditions, mTORC1 is activated and interacts with ULK1 and ATG13. mTORC1 activation leads to the phosphorylation of ULK1 and hampers autophagy. When the cells are confronted with starvation and other stress pressures, mTORC1 is inactive and accompanied by ULK1 dephosphorylation to form the ULK1 complex (which consists of ULK1, ATG13, FIP200, and ATG101) and initiates autophagy. Then, ATG21 and ATG24 will be combined into a phagophore [32]. Soon afterwards, ULK1 recruits and phosphorylates the VPS34 complex to activate it, and then, the VPS34 complex serves as signaling molecules to produce phosphatidylinositol-3-phosphate (PI3P) and recruit relevant proteins to facilitate the formation and expansion of phagophores.

During the formation and expansion of phagophores, ATG12 and LC3 will commence their pivotal function. The VPS34 complex will recruit ATG12, LC3, ATG5, ATG16, and other related proteins; under the effect of the E1-like enzyme and E2-like enzyme, ATG12 will combine with ATG5 and ATG16L1 and finally form the ATG12-ATG5-ATG16L1 complex, which can bind to the phagophore to promote its expansion. The ATG7-ATG3 ubiquitin-conjugating system is also crucial for the elongation of the phagophore through transforming LC3-I to LC3-II [33,34]. LC3-II functions as an anchor to autophagic proteins to promote the autophagy process.

Finally, under the execution of the monomer GTPase and endosomal sorting complex required for transport (ESCRT), mature autophagosomes can combine with lysosomes through the microtubule framework to form autolysosomes. At this time, various hydrolytic enzymes in lysosomes can hydrolyze and digest the contents, and transport them to the cytoplasm for recycling [35].

**The autophagic pathway can be summarized in several steps:** (a) The formation and extension of phagophores: mTOR signaling functions as a sensor contributing to autophagy. Under starvation and other stress pressures, the PI3K-Akt pathway and AMPK-ERK pathway inhibit the activity of mTORC1. The inhibition of mTORC1 promotes the dephosphorylation of ULK1, which contributes to the formation of the ULK1 complex and the initiation of autophagy (ULK1 recruits and phosphorylates the VPS34 complex to activate it, and VPS34 recruits relevant proteins to facilitate the formation and expansion of phagophores, not shown in the figure). ATG12 and the ATG7-ATG3 system will also commence their pivotal function. The VPS34 complex will recruit ATG12, LC3, ATG5, ATG16, and other related proteins; under the effects of the E1-like enzyme (ATG7) and E2-like enzyme (ATG10), ATG12 will combine with ATG5 and ATG16L1 and finally form the ATG12-ATG5-ATG16L1 complex, which can bind to the phagophore to promote its expansion. The ATG7-ATG3 ubiquitin-conjugating system is also crucial for the elongation of phagophores through transforming LC3-I to LC3-II. LC3-II functions as an anchor to autophagic proteins to promote the autophagy process. (b) Membrane nucleation and autophagosome maturation. (c) Autophagosomes fuse with lysosomes: mature autophagosomes combine with lysosomes through the microtubule framework to form autolysosomes. (d) The degradation of cargo by lysosomal acid proteases: hydrolytic enzymes in lysosomes hydrolyze and digest the cargo for recycling.

### 4.2. Gene Regulation of Autophagy

#### 4.2.1. ATG

ATG (autophagy-related protein) is a key executive factor in the autophagy process of cells. At present, more than 30 members have been found in the ATG family, which form complexes with each other and play a regulatory role in each process of autophagy [36]. They are primarily found in yeast, and their homologous proteins are also present in mammalian cells. The precise function of the individual ATG was delineated in the previous section.

#### 4.2.2. mTOR

mTOR is a class of serine/threonine protein kinase with a kinase domain at the C-terminal and can interact with a variety of proteins to form two different complexes: the mTORC1 complex and the mTORC2 complex. mTORC1 is sensitive to rapamycin and can participate in the regulation of ULK1 [37].

The PI3K-AKT-mTOR and AMPK-TSC1/2-mTOR signaling pathways, two upstream pathways of autophagy, have been studied. In the PI3K-AKT-mTOR signaling pathway, under nutritionally adequate conditions, phosphatidylinositol-3-kinase (PI3K) catalyzes PIP-generated PIP2 and PIP3, which can provide anchor points for signal transduction proteins [38]. The N-terminal of the serine/threonine protein kinase AKT contains the PH domain, which can bind closely to the 3-phosphate groups of PIP2 and PIP3 molecules, and then be activated and released into the cytoplasmic matrix and nucleus. Through phosphorylation, the protein is involved in tuberous sclerosis TSC1/2, its inhibition of G protein Rheb and activated protein kinase mTOR is removed [39]. mTOR can inhibit the kinase activity of ATG1, thereby inhibiting the occurrence of autophagy.

In the AMPK-TSC1/2-mTOR signaling pathway, adenosine monophosphate-activated protein kinase AMPK is also a serine/threonine protein kinase, which serves as an energy receptor that maintains metabolic homeostasis in cells [40]. The stimulation of environmental factors, such as hypoxia, can lead to the decrease in intracellular ATP and the increase in AMP, namely the AMP/ATP ratio increases; at this point, the catalytic subunit of AMPK is activated through the mediation of LKB1 [41]. It causes the activation of TSC2, which further leads to the decline in mTOR activity, and promotes the formation of autophagosomes, or AMPK can directly activate ULK1, thus activating autophagy.

#### 4.2.3. ULK1

ULK1 (unc-51-like autophagy activating kinase 1), which is present in mammalian cells, is also a protein kinase orthologue of Atg1 in yeast. As a key molecule of the autophagy initiation complex [42], ULK1 can directly regulate the formation of autophagosome and mediate the classic autophagy pathway, and the stability of the ULK1 complex with ATG13 and FIP200 is also important for the initiation of autophagy.

According to the study on autophagy signal transduction, mTORC1 can inhibit the activity of the ULK1 complex by phosphorylating ULK1 and ATG13 when the energy is sufficient [43,44], thus inhibiting the autophagy of cells. If the energy is insufficient, mTORC1 is inactive and ULK1 will be dephosphorylated and promote the formation of autophagosomes. It was found that, if the encoding gene of ULK1 was missing in mice, autophagy could not occur under any conditions [45]. If ULK1 was present but silent, autophagy was also significantly reduced. In a nutshell, ULK1 is indispensable for the autophagy of cells.

#### 4.2.4. Beclin-1

Beclin-1, which exists in mammalian cells, is a protein kinase orthologue of Atg6 in yeast [46]. Its structural domain includes BH3, central coiled coil region (CCD), and evolutionary conserved region (ECD). It is mainly involved in the process of phagophore nucleation and extension. The Beclin-1 regulatory network plays an important role in the regulation of autophagy in cells.

Firstly, the activated ULK1 complex directly phosphorylates the members of the VPS34 complex, like Beclin-1 at Ser, and activates lipase VPS34, type III PI3K in mammals [47]. Beclin-1 binds to VPS34 through its two structural domains, CCD and ECD. At the same time, by virtue of VPS34 binding to VPS15, it finally forms the VPS34-VPS15-Beclin-1 complex, which promotes the translocation of the autophagy-related proteins to autophagy initiation sites and increases the level of autophagy [47].

Secondly, the BH3 domain in Beclin-1 enables BCL-2 or BCL-XL to be recognized and combined with Beclin-1 to affect its activity. The autophagy level of the cell will be improved when the Beclin-1- BCL-2 complex is destroyed [46].

Finally, during the formation of the VPS34-Beclin-1 complex, there was also an UVRSG gene (UV radiation resistance-associated gene protein) involved in the formation of the VPS34-Beclin-1 complex [48], which is combined with Beclin-1 to increase the degree of interaction among various proteins in the complex, thus affecting the maturation and transportation of autophagic vesicles, and increases the level of autophagy. At the same time, ATG14, which is bound and phosphorylated by ULK1, can also promote the binding of this complex [49] and then participates in the extension of autophagic vesicles and regulates autophagy.

#### 4.2.5. p62

The ubiquitination junction protein p62 has multiple functional domains, and the absence of AMPK and ULK1 can lead to the abnormal accumulation of p62 [50], leading to the occurrence of mitophagy. The LIR and PB1 domains are more related to autophagy. The microtubule-associated protein 1 light chain 3 (LC3) protein, an orthologue of Atg8 in yeast, is of paramount significance in autophagy. During this process, the LC3-I and LC3-II proteins are mainly involved in mutual transformation and participate in the extension of autophagosomes. LC3 proteins bind to and interact with proteins through the LIR domain in p62 [51]. The other region PB1 can regulate autophagy through its own oligomerization.

#### 4.2.6. AMPK

AMP-activated protein kinase (AMPK) is a serine/threonine protein kinase complex that is involved in the positive regulation of autophagy pathways in cells. AMPK is sensitive to the changes in intracellular energy; an increase in the AMP:ATP ratio could lead to the activation of AMPK. At the same time, it can inhibit mTORC1 activity through phosphorylating its member RAPTOR or phosphorylating and activating ULK1 [52], promoting the formation of autophagosomes.

### 4.3. Role of Autophagy in Tumorigenesis and Cancer Treatment

The escaping from cell death procedure is one of the most remarkable characteristics of cancer cells. Among the various modalities of cell death, autophagy occupies an important place. The occurrence of autophagy is more context-dependent and supposed to be manipulated to cancer treatment. In the early stage of cell transformation and tumorigenesis, autophagy is proved to suppress tumor initiation, whereas it supports cancer cell growth and maintenance in established tumors. A study on breast cancer revealed that Beclin 1 is expressed at a low level in human breast epithelial carcinoma cell lines and tissue, and the forced expression of Beclin 1 in breast cancer cells could increase autophagy activity and inhibit the cancer cells’ proliferation and tumorigenesis capacity in nude mice [53]. When it comes to Ras-driven tumors, scientists found the oncogene Ras was capable of up-regulating autophagy to provide nutrients for tumor proliferation and development [54]. There are also a myriad of studies indicating the importance of epigenetic regulation to autophagy in cancer. Aberrant DNA hypermethylation contributed to the lower expression of Beclin 1 and enforced the autophagy pathway to facilitate the development of invasive ductal breast cancer (IDC) [55]. In hepatocellular carcinoma (HCC), P300/CBP-associated factor (PCAF) induced the autophagy of HCC cells and elicited cell death [56].

By reason of the foregoing, targeting autophagy pathway offers a unique window of therapeutic potential, and relevant clinical drugs, such as CQ and HCQ [57], are proven to have great potential in inhibiting autophagy and pharmacological intervention in cancer.

## 5. Pyroptosis

### 5.1. Signal Transduction of Pyroptosis

Pyroptosis is an inflammatory type of programmed cell death that was coined in 2001 and relies on caspase-1 [58]. Through caspase-dependent activation, the cleavage and oligomerization of the Gasdermin family occurs, resulting in cell perforation-mediated cell pyroptosis [59]. The morphology of the cell is characterized by the osmotic lysis of the cell until the cell membrane ruptures, causing the release of the cell contents (inflammatory factors and lysosomes), which activates a strong inflammatory response. As a current research hotspot, it has been found that there are two ways to carry out pyroptosis: the caspase-1-dependent canonical pathway and caspase-4/5/11-dependent non-canonical pathway [60].

#### 5.1.1. Classic Pyroptotic Pathway

The inflammasome is a complex of several proteins and has cytoplasmic pattern recognition receptors (PRRs), which are also involved in the assembly of inflammasomes and serve as a key part of the natural immune system. It recognizes and responds to pathogen-associated molecular patterns (PAMPs) or damage-associated molecular patterns (DAMPs); then, pro-caspase-1 is recruited to cleave and activate it [61]. Since the caspase-1-dependent inflammasomes NLRP1, NLRP3, NLRC4, and AIM2 are called classical inflammasomes, their causative pyroptotic pathways are called classical pyroptotic pathways [62].

**Figure 3:**
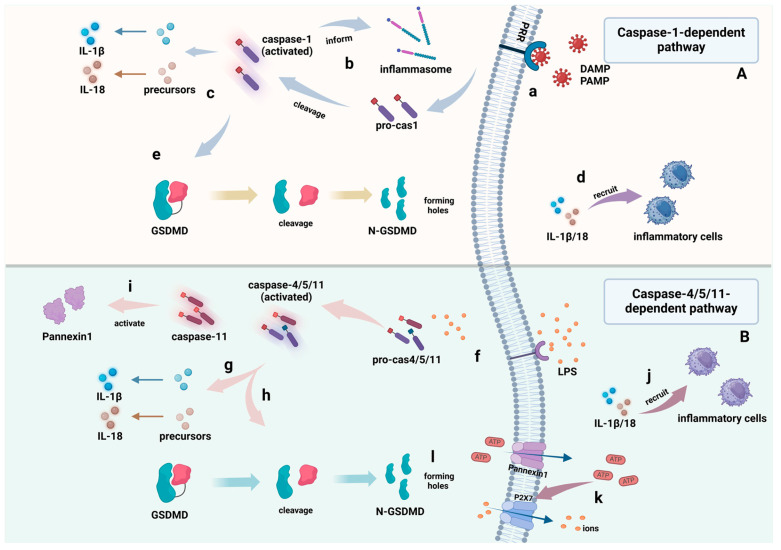
Pathways of pyroptosis (created with BioRender.com).

As shown in Figure 3, the classical pyroptosis pathway is stimulated by the signals of pathogens, such as bacteria or viruses, and the PRRs in cells act as receptors to recognize these signals. Some inflammasome sensors containing the CARD domain can directly recruit pro-caspase-1 to form the inflammasome. In contrast, inflammasome sensors without the CARD domain need to activate pro-caspase-1 through the binding of the apoptosis-related particle protein (ASC) to pro-caspase-1 to form a multiprotein complex termed inflammasome. Activated caspase-1 can cut interleukin-precursors of IL-1β and IL-18 to form mature cytokines, which are released into the extracellular environment, recruit inflammatory cells, and trigger an inflammatory response [63]. At the same time, Gasdermin D was also cleaved into the N-terminal pore-forming domain (PFD) and the C-terminal repressor domain (RD), and PFD formed non-selective holes in the membrane, which induced cell membrane perforation, caused cell swelling and rupture, released contents, and caused cell paroxysmal death [60].

There are two forms of pyroptosis: the caspase-1-dependent canonical pathway and caspase-4/5/11-dependent non-canonical pathway. **In the caspase-1-dependent canonical pathway (Part A)**, (a) PRRs in the cell membrane recognize and interact with PAMP/DAMP, (b) then facilitate the activation of pro-caspase-1 to caspase-1 and the formation of inflammasomes. (c) Activated caspase-1 can cut interleukin-precursors of IL-1β and IL-18 to form mature cytokines. (d) Mature IL-1β and IL-18 are released into the extracellular environment and recruit inflammatory cells, thus triggering and inflammatory response. (e) Activated caspase-1 can also contribute to the cleavage of GSDMD, and the PFD domain of GSDMD forms non-selective holes in the cell membrane, causing cell swelling and rupture.

**In the caspase-4/5/11-dependent non-canonical pathway (Part B)**, (f) LPS enters the cytoplasm to perform pro-caspase-4/5/11 oligomerization and activation. Activated caspase-4/5/11 contributes to the (g) maturation of IL-1β and IL-18, (h) cleavage of GSDMD, (i) and activation of Pannexin-1. Then, (j) mature IL-1β and IL-18 are released into the extracellular environment and recruit inflammatory cells for inflammatory responses; (k) Pannexin-1 translocates to the membrane and mediates the release of ATP and other small ions, and ATP release leads to the opening of the P2X7 channel, which leads to the further release of some ions; and (l) the PFD domain of GSDMD forms non-selective holes in the cell membrane, causing cell swelling and rupture.

#### 5.1.2. Non-Classical Pyroptotic Pathways

The non-classical pyroptotic pathway is triggered by its host inflammatory stimulation factor lipopolysaccharide (LPS), with the CARD of caspase-4/5/11 directly binding to LPS after it enters the cytoplasm to perform its own oligomerization and activation; then, it cuts GSDMD at the junction between the N-terminal and C-terminal domains, inducing GSDMD activation and cell membrane perforation, causing cell swelling until the cell membrane ruptures and releases its contents, inducing pyroptosis. At the same time, caspase-4/5/11 can also induce the maturation and secretion of IL-1β and IL-18 by the NLRP3/caspase-1 pathway [64] to produce an inflammatory response.

In addition, activated caspase-11 was observed to cause to the cleavage and modification of Pannexin-1, a channel that controls the passage of small molecules in and out of the membrane, releasing a large amount of ATP and some other small ions. Instead, ATP release leads to the opening of the Pannexin-1-dependent ATP channel P2X7, leading to the further release of some ions [65], resulting in the swelling and rupture of cells, thereby mediating cell pyroptosis.

Recent studies have found that killer lymphocytes can also induce the pyroptosis of tumor cells, demonstrating that pyroptosis can be occur without caspase activation and it is a potential pathway for tumor medication. The mechanism shows that GSDME in tumor cells is able to recruit killer lymphocytes and activate caspase-independent pyroptosis by directly cleaving GSDME [66].

### 5.2. Gene Regulation of Pyroptosis

#### 5.2.1. Gasdermin Family Proteins

The Gasdermin family protein (GSDM) consists of six members, GSDMA, GSDMB, GSDMC, GSDMD, GSDME, and DFNB59, in humans and five proteins, Gsdma, Gsdmc, Gsdmd, Dfna5, and Dfnb59, in mice [67]. GSDMD was the first to be identified as an executive molecule of pyroptosis [59], and subsequent studies have found that GSDME proteins in this family can also regulate pyroptosis after being sheared and activated by caspase-3 [68,69]. Gasdermin family proteins show membrane perforation activity. They are activated after being cleaved by inflammatory caspases in cells, and their N-terminal domains are oligomerized to form pores in the cell membrane [70]. GSDMD-N mediates cell pyroptosis: Firstly, GSDMD-N undergoes an oligomerization process to form an oligomer; then, it translocates to the cell membrane and forms non-selective pores to facilitate pyroptosis [70].

#### 5.2.2. GPX4

Glutathione peroxidase 4 (GPX4) is a recently discovered negative regulator of pyroptosis. Sepsis is a disease caused by pathogen infection and when the host’s autoimmune response is disordered; pyroptosis plays an important role in this process. Studies have found that the antioxidant enzyme GPX4 can negatively regulate the septic death of macrophages during the process of GSDMD-N-mediated pyroptosis [71]. GPX4 is an inhibitory protein in the lipid peroxidation process, which can inhibit the activity of caspase-11, so that GSDMD cannot be cleaved to produce an active N-terminal or impede pyroptosis in macrophages involved in sepsis [72].

### 5.3. Aiming at Pyroptosis in Cancer

Considerable research has focused on pyroptosis and uncovered its indispensable role in cancer development and related pharmacological therapy. In hepatitis B virus-related hepatocellular carcinoma (HBV-HCC), a study [73] used the pyroptosis phenotype to stratify HCC patients into two subtypes and found that pyroptosis had a strong correlation with patients’ prognosis, the tumor immune microenvironment, and immunotherapy. The pyroptosis level also influences the anti-PD-L1 treatment. GSDME has been regarded as a potential tumor suppressor gene [74]. In research on breast cancer, GSDME presented a higher methylation level in breast cancer samples compared to normal breast samples and blunted cells’ pyroptosis [75]. This was accompanied by facilitating the invasiveness of breast cancer cells [76]. In the development of drugs to treat leukemia, the natural small-molecule pyridoxine (vitamin B6) was confirmed to induce pyroptosis in the AML cell line THP-1 and showed promise as a possible drug for AML treatment [77]. Pyroptosis and the gasdermin family play pivotal roles in tumorigenesis as well as progression; thus, targeting this pathway shows the forceful potential of individualized tumor therapy.

## 6. Ferroptosis

### 6.1. Signaling of Ferroptosis

Since being reported in 2012 [78], ferroptosis has been attracting considerable interest. Ferroptosis is a new type of iron-dependent programmed cell death that is caused by iron-dependent peroxidation. The process is mainly dependent on the regulatory cell death (RCD) caused by lipid peroxidation induced by iron and reactive oxygen species (ROS) [79]. The main mechanism of ferroptosis is due to the metabolic disturbance of lipid oxides. Under the action of lipoxygenase (LOXs) or divalent iron, unsaturated fatty acids, which are highly expressed on the cell membrane, are catalyzed to produce a large amount of lipids, and liposome peroxidation destroys the intracellular redox balance, thus inducing cell death [80].

**Figure 4:**
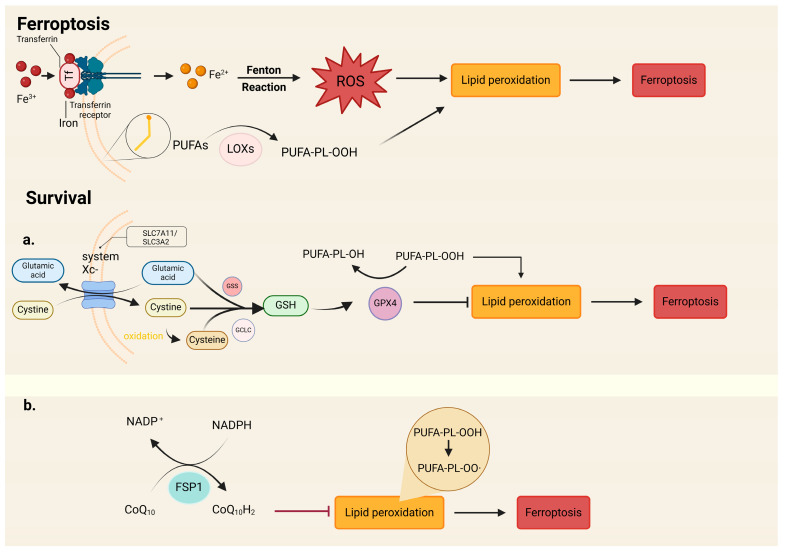
Pathways of cell iron death (created with BioRender.com).

As shown in Figure 4, there are multiple pathways for ferroptosis, but in essence, all of them are caused by the accumulation of reactive lipids on membrane lipids through direct or indirect influence on the activity of glutathione peroxidase (GPX4). In addition, the pathway directly inhibits GPX4 so that it cannot catalyze the reduction of H_2_O_2_ and hydroperoxides, resulting in the increase in iron-dependent ROS, leading to ferroptosis. The classical pathway is induced by inhibiting the Na^+^-dependent cystine/glutamate reverse transporter system (System Xc-) [81]. System Xc- is made up of SLC7A11 and SLC3A2 disulfide bond dimers. Through the channels of glutamic acid from the cell, it can be transferred to the extracellular environment and cystine from the extracellular to the intracellular environment and be reduced into cysteine in glutathione (GSH). The synthesis of GSH has antioxidant effects and can protect cells against oxidative stress damage. When System Xc- is inhibited, GSH synthesis is reduced, and GSH-dependent GPX4 activity is down-regulated, leading to the accumulation of lipid reactive oxygen species and ferroptosis [82].

**The process of ferroptosis:** Fe^3+^ in the blood circulation binds to transferrin and is reduced to Fe^2+^ after entering the cell through the transferrin receptor. Fe^2+^ undergoes the Fenton reaction and produces considerable amounts of ROS. Under the action of lipoxygenases (LOXs) and ROS, the unsaturated fatty acids are catalyzed to produce many lipids, and lipid peroxidation destroys the intracellular redox balance, thus inducing ferroptosis.

**In survival cells:** (a) System Xc-, which is composed of SLC7A11 and SLC3A2, is responsible for substance transport (glutamic acid/cystine). Glutamic acid, cystine, and oxidative cystine (cysteine) are reduced into GSH; the synthesis of GSH can protect cells against oxidative stress damage. GPX4 is GSH-dependent and is crucial for reducing lipid peroxides, thereby reducing oxidative stress damage. (b) The ferroptosis inhibitor protein FSP1 can prevent lipid peroxidation, and the reduced form of CoQ10 can capture the lipid peroxidation free radicals that can mediate lipid peroxidation. Then, FSP1 through NADPH can catalyze the restoration of CoQ to a reduced form.

### 6.2. Gene Regulation of Ferroptosis

#### 6.2.1. p53

As a tumor suppressor gene, p53 is involved in the regulation of many mechanisms of programmed cell death. In addition to these two classical pathways, there are several other pathways of ferroptosis. One of these pathways is p53-mediated ferroptosis. p53 inhibits the expression of SLC7A11, a key component of the cystine/glutamate reverse transporter [83], and inhibits cell transport to cystine, resulting in reduced glutathione synthesis, decreased antioxidant capacity of cells, increased concentration of lipid reactive oxygen species, and an increase in cell sensitivity to ferroptosis.

#### 6.2.2. CoQ REDOX Enzyme FSP1

The ferroptosis inhibitor protein FSP1, formerly known as mitochondrial apoptosis inducer 2 (AIFM2), is a glutathione-independent ferroptosis inhibitor that is mediated by ubiquinone CoQ10. Studies have found that FSP1 can prevent lipid peroxidation, and the reduced form of CoQ10 can capture the lipid peroxidation free radicals that can mediate lipid peroxidation; then, FSP1 through NADPH can catalyze the restoration of CoQ to a reduced form [84]. Cells without FSP1 are also more sensitive to ferroptosis. Moreover, FSP1 has a synergistic effect with GPX4 and GSH, which can jointly play a role in inhibiting ferroptosis.

### 6.3. Ferroptosis Regulation in Cancer

Emerging evidence has shown that ferroptosis plays a significant role in cancer. A study analyzed gastric cancer (GC) and discovered that hypoxia-induced lncRNA could affect ferroptosis resistance in GC cells by a m6A methylation-dependent mechanism [85]. In pancreatic ductal adenocarcinoma, CPEB1 serves as a key ferroptosis regulator whose silence promotes the translation of p62/SQSTM1 and NRF2 stability, thus leading to the activation of anti-ferroptosis genes [86]. The transferrin receptor TFRC is highly expressed in bladder cancer with diagnostic and prognostic value, and the repressed expression of TFRC could inhibit ferroptosis induced by Erastin in bladder cancer cells [87]. In colorectal cancer therapy, researchers tried to apply a supramolecular nanoreactor (named DOC@TA-Fe^3+^) to cancer treatment, which led to a good progress. DOC@TA-Fe^3+^ could escape from the lysosomes, trigger the Fenton reaction, and induce ferroptosis in colorectal cancer cells. The result showed that the nanoreactor has a good application prospect in cancer therapy, and targeting ferroptosis is also a satisfactory pharmacological intervention [88].

## 7. Cuproptosis

As a fundamental trace element to maintain the body’s physiological equilibrium, copper is obtained by the diet, and its absorption and export is regulated by the corresponding proteins in cells. An insufficient copper intake can impede body growth, and an excessive copper intake can cause oxidative stress, cell death, and tissue damage.

There are two ionic states of copper in the body: cupric state (Cu^2+^) and cuprous state (Cu^+^). Cu^+^ is the main form present in cells. Copper acts as a necessary cofactor for mediating many basic cellular functions of enzymes. At the same time, breaking the normal level of copper ion concentration can induce oxidative stress and cytotoxicity [89,90]. Cuproptosis is a novel form of cell death that was proposed by Tsvetkov [91] and is characterized by being copper-dependent and regulating mitochondrial respiration. During cuproptosis, copper directly is combined with the lipoylation proteins in the tricarboxylic acid (TCA) cycle, leading to the accumulation of fatty acylated proteins and the subsequent loss of iron–sulfur cluster proteins, leading to protein toxic stress and eventually to cell death [92].

### 7.1. Signaling of Cuproptosis

**Figure 5:**
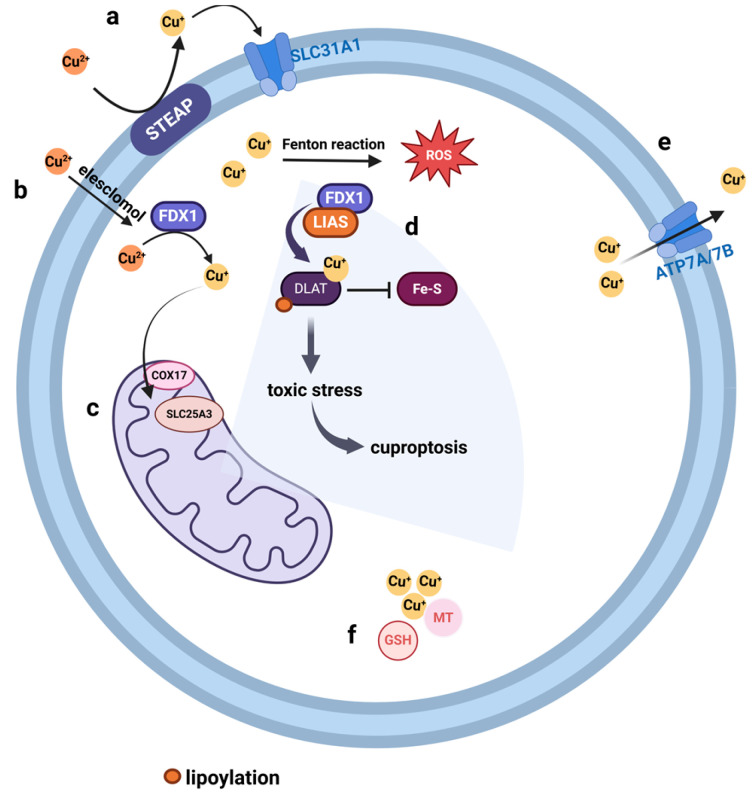
Pathways of cuproptosis (created with BioRender.com).

Copper accumulation is related to cuproptosis. First, copper ions generate a large amount of reactive oxygen species (ROS) through the Fenton reaction, which induces DNA damage and lipid peroxidation [93]. Second, copper ions inhibit cell protease activity and cell proliferation by inhibiting the ubiquitin proteasome system [94]. In addition, copper ions bind to fatty acylated proteins, such as pyruvic acid α- ketoglutaric acid, branched chain keto acid dehydrogenase, and glycine cleavage system; induce fatty acylated protein aggregation; inhibit mitochondrial metabolic function; and promote cell cuproptosis [95]. Ferredoxin1 (FDX1) and lipoic acid synthase (LIAS) play an important regulatory role in this process [96]. The basic mechanism of cuproptosis is shown in Figure 5.

Copper can enter cells in two ways: (a) After being reduced to Cu^+^ by STEAP, Cu^+^ is absorbed by cells through the membrane protein SLC31A1; (b) elesclomol transfers Cu^2+^ into cells, and then, intracellular Cu^2+^ is reduced to Cu^+^ by FDX1. (c) In the cell, COX17 carries Cu^+^ from the cytoplasm to the mitochondrial membrane space, and then, Cu^+^ can be transported from the mitochondrial membrane space to the mitochondrial matrix through SLC25A3. (d) FDX1 and LIAS promote the acylation of dihydrolipoamide S-acetyltransferase (DLAT) and reduce the iron–sulfur cluster proteins, inducing cuproptosis. Furthermore, copper ions generate a large amount of ROS through the Fenton reaction, which induces DNA damage and lipid peroxidation. (e) And the efflux of copper is mediated by ATP7A/7B. (f) Intracellular proteins involved in the binding and storage of copper, such as metallothionein (MT) and glutathione (GSH), bind to intracellular Cu^+^ to prevent it from damaging cells.

### 7.2. Gene Regulation of Cuproptosis

#### 7.2.1. FDX1

Ferredoxin1 (FDX1), which is a small molecule protein containing iron atoms and inorganic sulfides with an electron transfer effect [97], can transfer electrons from NADPH to mitochondrial cytochrome P450 and participates in the metabolism of various substances [98]. Research has found that FDX1 can encode a reductase, which is known to reduce Cu^2+^ to a more toxic form of Cu^+^ and become a direct target for enols [99]. FDX1 promotes the acylation of dihydrolipoamide S-acetyltransferase (DLAT) and reduces iron–sulfur cluster proteins and induces cell death by cuproptosis. The internal balance of copper depends on its transporters, and upsets in this internal balance can lead to copper deficiency [100]. FDX1 is considered as a pivotal regulator of cuproptosis, since FDX1 depletion leads to the complete loss of protein lipoylation, a marked decrease in cellular respiration, accumulation of pyruvate and α-ketoglutarate, reduction in succinate, and stabilization of Fe-S cluster proteins. Altogether, excessive copper promotes the aggregation of lipoylated TCA cycle proteins and destabilization of Fe-S cluster proteins, both of which are mediated by FDX1, consequently resulting in cuproptosis [101].

A pan-cancer study that performed a systematic characterization of cuproptosis-related genes across more than 9000 samples of 33 types of cancer verified the relation between cuproptosis genes and cancer progress, and found that kidney renal clear cell carcinoma (KIRP) was the most affected cancer whose survival was the most associated with cuproptosis genes [102]. The high expression of FDX1 was associated with a low survival risk in KIRP. In addition, cuproptosis genes are also involved in the activation of other cancer pathways and interact with cancer-associated miRNAs. Another study made clear that FDX1 has a unique expression pattern across various cancers and it might be a potential predictor of treatment effects for tumor patients. Additionally, the genes involved in the tricarboxylic acid (TCA) cycle are particularly affected [101]. In colon adenocarcinoma (COAD), FDX1 expression has a strong correlation with tumor immunity, especially CD8^+^ T cells and CD4^+^ T cells, and the pathogenesis of COAD [103]. Emerging research proved METTL16-mediated m6A modification on FDX1 mRNA was critical for cuproptosis in gastric cancer (GC), and METTL16 lactylation significantly promoted the therapeutic efficacy of the copper ionophore-elesclomol [104]. In summary, FDX1-oriented cuproptosis regulates the propagation of diverse cancers; hence, targeting FDX1 is a strong candidate for cancer treatment.

#### 7.2.2. Other Protein Families

Some protein family members also play vital roles in intracellular copper ion absorption, transport, and export, like Steap proteins, solute carrier family member SLC31A1, and P-type ATPases. Steap proteins are located in the duodenum and can convert copper from cupric state to cuprous state. Research based on the transient expression of Steap proteins in HEK cells demonstrated their indispensable function in cellular copper assimilation [105]. As the important copper transporter, SLC31A1 has the function of absorbing copper from the daily diet into the cell membrane. A study confirmed the findings of Qu, P et al. (2023), which found that the expression of SLC31A1 showed a very distinct pattern in different cancer species. And SLC31A1 was related to patients’ prognosis as well as immune cell infiltration in several cancers [106]. Recent research has shown that advanced glycosylation end products (AGEs) could contribute to cuproptosis and mitochondrial dysfunction mediated by the up-regulation of SLC31A1 in cardiomyocytes; related pathways may be a potential therapeutic target specific to diabetic cardiomyopathy (DCM) [107]. In the field of intervertebral disc degeneration (IDD), researchers indicated that ATP7A, an efflux pump of cuproptosis, was up-regulated by oxidative stress followed by the increase in TCA cycle-related protein aggregation and cuproptosis [108].

### 7.3. Epigenetic Regulation of Different Types of Cell Death

Epigenetic modification is of great significance in regulating biological characteristics as well as participating in multiple physiological and pathological processes without altering DNA sequence. It consists of histone modification, DNA methylation, RNA methylation, noncoding RNA regulation, etc. Considerable research has indicated that different forms of epigenetic modification take part in the onset and progression of cancer. Breaking the epigenetic balance can seriously affect the normal development of the body and mediate the pathological process. There are also numerous studies that have proven the important regulatory mechanism of epigenetics in different modalities of cell death. So, understanding how cell death is regulated in cancer from an epigenetic perspective is also important for understanding the disease and developing treatment strategies. In view of this, we also discussed the significance of different epigenetic modification in cancer cell death and summarize them in Table 2.

### 7.4. Targeting Cell Death as Cancer Therapy

**Figure 6:**
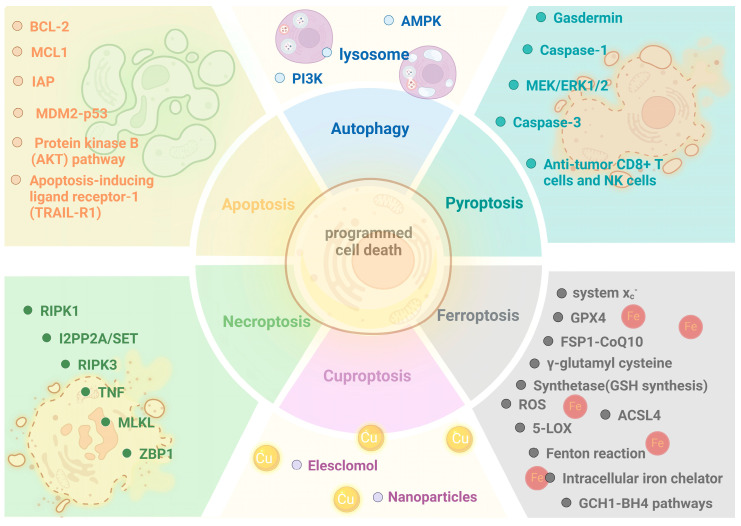
Targeting cell death in cancer.

Due to the importance of cell death in cell biology and diseases, a large number of trials and experiments have been put into motion to develop drugs and inhibitors targeting the abnormal cell death program in cancer. There are some key factors that play a decisive function in cell death and their dysfunction leads to pathological progression; they are always regarded as the targets with most potential in drug design. According to the published research, we summarize the existing drugs and inhibitors, which target core factors of cell death in cancer (Table 3). Among the cell death modalities, drug therapies targeting cuproptosis in cancer focus on nanodelivery technology [202,203,204]. We also drew a schematic summary of the protocols for targeting cell death in cancer medication (Figure 6).

The targets for the treatment of several classical programmed cell death include some important signaling pathways, enzymes, and immunocytes. This figure summarizes these targets.

## 8. Summary and Prospect

Evidence suggests that programmed cell death is among the most important factor for the healthy operation of cells and even the body, and the timely removal of anomalous cells is essential to maintain the stability and balance of the body. In recent years, with increasingly rapid advances in the field of programmed cell death, a large amount of cell death modalities, such as mitotic catastrophe, entosis, netosis, parthanatos, and other death modes, have been discovered. Although studies in recent decades have provided important information on programmed cell death, many specific regulatory mechanisms remain unclear. Meanwhile, the mechanism of diverse programmed cell death in organism evolution, tissue homeostasis, organ development, and disease occurrence has not been fully understood. Many studies have shown that the occurrence, development, and treatment of tumors are closely related to programmed cell death. Therefore, the regulatory role of programmed cell death and its interplay with cancer are still the focus of current research. The modes of cell death are not only limited to gene regulation, but also extending to epigenetic regulation, etc. Therefore, exploring the molecular mechanisms underlying cancer development can provide a solid theoretical and experimental basis for the diagnosis, treatment, prevention, and drug development of diseases. As for targeting the death pathway of tumor cells, there also have some existing problems. For instance, tumor cells become resistant to the drugs; the way cells die may change to another form under specific stress conditions; which death pathway cancer cells tend to enter is context-dependent. Therefore, we still have considerable work to conduct to unveil the mystery of cell death and formulate feasible clinical management strategies.

## Figures and Tables

**Table 1:** Modalities of cell death and their characteristics.

Type	Morphology	Biochemical Characteristics	Inducement
Apoptosis	membrane blebbing; cell shrinkage; condensation of chromatin; fragmentation of DNA; absence of inflammatory response	activation of caspases	anticancer drugs; gamma and ultraviolet irradiation; deprivation of survival factors; cytokines
Autophagy	membrane extension; de novo formation of cytosolic vesicles; lack of chromatin condensation	caspase-independent; LC3 lipidation; formation of autophagosome; elevated autophagic flux and lysosomal activity	starvation and other stresses
Pyroptosis	DNA fragmentation; chromatin condensation; pore formation; cell swelling and osmotic lysis; plasma membrane rupture	inflammasome assembly; GSDM cleavage; release proinflammatory cytokines and other cellular contents	invading pathogens (pathogen-associated molecular patterns (PAMPs)); endogenous pathogens (damaged-associated molecular patterns (DAMPs)); bacteria (lipopolysaccharide (LPS))
Necroptosis	cell swelling; pore formation; rupture of plasma membranes; moderate chromatin condensation	caspase-independent; RIPK1/RIPK3-mediated phosphorylation of MLKL; necrosome; infammation and immune responses	viral infection; activation of death receptors/Toll-like receptors/cytosolic nucleic acid sensors
Ferroptosis	loss of plasma membrane integrity; cytoplasmic swelling; swelling of cytoplasmic organelles; membrane shrinkage with increased membrane density and a reduced number of or loss of mitochondrial cristae; normal nuclear size	ROS-dependent; iron accumulation; lipid peroxidation	Erastin; intracellular iron perturbations; oxidative stress; activation of mitochondrial voltage-dependent anion channels and mitogen-activated protein kinases; up-regulation of endoplasmic reticulum stress; inhibition of cystine/glutamate antiporter
Cuproptosis	mitochondrial shrinkage; rupture of mitochondrial membrane	copper accumulation; aggregation of lipoylated dihydrolipoamide S-acetyltransferase (DLAT); loss of iron–sulfur cluster (Fe-S) proteins; increased level of ROS	elesclomol; lipoylated dihydrolipoamide S-acetyltransferase (DLAT)
Disulfidptosis	F-actin contraction and detachment from the plasma membrane; collapse of cytoskeleton proteins; cell shrinkage	disulfide stress; redox-sensitive protein disulfide binding; high expression of SLC7A11; formation of aberrant disulfide bonds between actin cytoskeleton proteins	glucose starvation; high cystine uptake; NADPH-depleting cytosolic environment

**Table 2:** Epigenetic regulation of cell death in cancer.

Cell Death Modality	Epigenetic Modification	Cancer Type	Description	Reference
Apoptosis	Histone modification	Non-small cell lung cancer (NSCLC)	Histone deacetylases SIRT6 impacted the modulation of antioxidant and redox signaling and apoptosis induction.	[109]
			Magnolol and polyphenol mixture (PM) derived from Magnolia officinalis exhibited remarkable anti-tumor activities as potential inhibitors of class I HDACs and induced tumor cell apoptosis partially by epigenetically activating DR5.	[110]
		Atypical meningioma (AM)	Certain apoptosis-associated factors were associated with the recurrence of AMs and regulated by histone 3 lysine methyltransferase.	[111]
		Endometrial cancer	PRMT6 regulated genomic regions related to interferons and apoptosis through histone modifications.	[112]
		Colorectal cancer (CRC)	WHSC1 regulated BCL2 gene through the H3K36 dimethylation level and protected colon cancer cell against apoptosis.	[113]
			N-terminal acetyltransferases (NATs) Naa40 inhibited the mitochondrial caspase-9-mediated apoptotic cascade.	[114]
		Gastric cancer (GC)	Anti-tumorigenic agent CIL-102 induced apoptosis in human gastric cancer by the H3K4 trimethylation of TNFR1 and TRAIL proteins.	[115]
			(-)-epigallocatechin-3-gallate (EGCG) impeded cancer progression by apoptosis induction, as well as the inhibition of cell proliferation and histone deacetylase.	[116]
		Hepatocellular carcinoma (HCC)	Histone methyltransferase G9a allowed DNA-damaged hepatocytes to escape p53-induced apoptosis by silencing Bcl-G (a pro-apoptotic Bcl-2 family member).	[117]
		Osteosarcoma	Histone methyltransferase NSD2 promoted the transcription of genes associated with the negative regulation of apoptotic signalling pathways and regulated the expression of the apoptosis regulatory proteins BCL2 and SOX2 through the ERK and AKT pathways.	[118]
	DNA methylation	Schwannoma	The promoter methylation of gene-encoding apoptosis-associated speck-like protein containing a caspase recruitment domain influences the activation of endogenous caspase-9 and caspase-3.	[119]
		Renal cell carcinoma (RCC)	DNMT3B silenced the gene that encoded the component of mitochondrial complex III and affected Cyt c release.	[120]
		Ameloblastoma	The transcription of the apoptosis-related gene BCL2L11 was possibly regulated by promoter DNA methylation.	[121]
		Rectal cancer	The methylation status of the promoter regions of key apoptosis genes correlated with apoptosis and the survival of rectal cancer patients.	[122]
		Glioblastoma multiforme (GBM)	Dnmts coregulated apoptosis-associated genes and dictated whether glioma harbors the apoptosis evasion phenotype.	[123]
		Bladder cancer (BCa)	The methylation of apoptosis-associated genes was significantly associated with tumor staging and grading, and methylation markers are promising tools for the noninvasive detection of bladder cancers.	[124]
	Noncoding RNA	Ovarian cancer	LncRNA DUXAP8 regulated the apoptosis of ovarian cancer cells by targeting miR-590-5p.	[125]
		Endometrial carcinoma (EC)	LncRNA EIF1AX-AS1 markedly inhibited EC cell proliferation and promoted apoptosis.	[126]
		Oral squamous cellular cancer (OSCC)	The LncRNA CEBPA-DT/CEBPA/BCL2 axis participated in the resistance to chemotherapy drug cisplatin through cell apoptosis.	[127]
		GC	TM4SF1-AS1 promoted stress granule formation and inhibited apoptosis in GC cells by sequestering RACK1, an activator of the stress-responsive MAPK pathway.	[128]
			The site-specific methylation of lncRNAs enhanced the stability of PSMA3-AS1 and MIR22HG to suppress apoptosis of gastric cancer stem cells via the PSMA3-AS1-miR-411-3p- or MIR22HG-miR-24-3p-SERTAD1 axis.	[129]
		NSCLC	SNHG6 promoted proliferation and inhibited apoptosis in NSCLC by regulating miR-490-3p/RSF1 axis.	[130]
		Prostate cancer (PCa)	LncMEG3 inhibited PCa proliferation and promoted apoptosis through the disruption of the miR-9-5p-mediated inhibition of NDRG1.	[131]
Autophagy	Histone modification	PCa	KDM4B activated autophagy by regulating the Wnt/β-catenin signaling and contributed to castration-resistant prostate cancer.	[132]
		NSCLC	The inhibition of EHMT2, a histone methyltransferase of histone H3 lysine 9, effectively induced cell death in NSCLC cells through altering cholesterol metabolism-dependent autophagy.	[133]
		GC	The inhibitor of EZH2 and EGFR exerted an effect on tumor growth inhibition through inducing autophagy.	[134]
		CRC	EZH2 bonded to the promoters of the negative regulators of the MTOR and modulated subsequent MTOR pathway-related events, including the inhibition of autophagy.	[135]
			JMJD2B regulated autophagy in CRC cells through LC3B as well as intracellular amino acid levels under glucose deprivation, so as to influence the survival of CRC cells.	[136]
	DNA methylation	Breast caner	The H19/SAHH/DNMT3B axis regulated the methylation level of Beclin1 and then contributed to inducing autophagy.	[137]
		Acute lymphoblastic leukemia (ALL)	High-throughput screens uncovered the autophagy-related gene ATG16L2 was associated with a poorer prognosis in childhood ALL.	[138]
		Ovarian cancer	The methylation status of ATG4A impacted the stem properties of ovarian tumor-initiating cells, and the hypomethylation of ATG4A predicted a poor prognosis for ovarian cancer patients.	[139]
		GC	Methylation status influenced the expression level of MAP1LC3Av1, which is essential for autophagy as well as gastric carcinogenesis.	[140]
	Noncoding RNA	Liver cancer	The high expression of miR-638 led to an increase in autophagosomes and autolysosomes through an increase and decrease in the expressions of LC3B-II and Beclin-1 proteins, respectively.	[141]
		Cholangiocarcinoma (CCA)	MiR-124 led to autophagic flux by the EZH2-STAT3 signaling axis.	[142]
		HCC	MiR-30a directly targeted the autophagy-related protein Beclin 1 and Atg5 and mediated autophagy activity.	[143]
		Gastrointestinal stromal tumors (GISTs)	MiR-30a was correlated with imatinib sensitization by the regulation of cell autophagy mediated by Beclin-1.	[144]
		Acute myeloid leukemia (AML)	MiR-143 inhibits autophagy in cytarabine-treated AML cells by directly targeting autophagy-related proteins (ATG7 and ATG2B).	[145]
Pyroptosis	Histone modification	CRC	HDAC2 suppressed the NLRP3 transcription as well as GSDMD-mediated pyroptosis by inhibiting the formation of the H3K27ac/BRD4/p-P65 complex.	[146]
		GC	The high expression of lysine demethylase ALKBH4 inhibited GSDME activation by inhibiting H3K4me3 histone modification and promoted the proliferation of gastric cancer cells.	[147]
		Melanoma/Lung carcinoma	MLL4 ablation attenuated the expression of the RNA-induced silencing complex (RISC) and DNA methyltransferases through decommissioning enhancers/super-enhancers, which consequently led to the transcriptional reactivation of the double-stranded RNA (dsRNA) interferon response and gasdermin D (GSDMD)-mediated pyroptosis, respectively.	[148]
		Multiple myeloma (MM)	PRMT5 regulates cell pyroptosis by silencing CASP1 in multiple myeloma.	[149]
		RCC	BRD4 exerted an anti-tumor effect in RCC by activating the NF-κB-NLRP3-caspase-1 pyroptosis signaling pathway.	[150]
		Glioblastoma	The usage of LSD1 inhibitor inhibited the proliferation of glioblastoma cells and induced their pyroptosis.	[151]
	DNA methylation	Breast cancer	Breast cancer samples showed a higher DFNA5 methylation in the putative gene promoter; DFNA5 methylation showed strong potential as a detection and prognostic biomarker for breast cancer.	[75]
		Clear cell renal cell carcinoma (ccRCC, KIRC)	The DNA methylation levels of GSDMA/B/D/E were decreased in ccRCC patients, and the high expression of GSDME indicated a poor overall survival and relapse-free survival.	[152]
		Breast cancer /CRC/GC/HCC	ZDHHC1, which is frequently silenced in cancer cells, played a role in promoting cell pyroptosis by enhancing oxidative stress and endoplasmic reticulum stress.	[153]
		Uveal melanoma/Lower grade glioma/Kidney renal clear cell carcinoma	Hypomethylation led to the high expression of pyroptosis-related genes in uveal melanoma, lower grade glioma, and kidney renal clear cell carcinoma, suggesting a poor prognosis.	[154]
		HCC	The hypomethylation of pyroptosis-related genes (PRGs) was associated with a poor prognosis of HCC. The gene body hypomethylation of PRGs is a promising biomarker for early HCC detection.	[155]
	Noncoding RNA	Lung adenocarcinoma (LUAD)	The knockdown of circPIBF1 significantly enhanced the expression of pyroptosis-related factors and suppressed LUAD cell growth.	[156]
		CRC	The inhibition of miR-15a increased inflammatory cytokines, activated caspase-1 inflammasome, and increased Gasdermin D, an effector of pyroptosis.	[157]
		EC	HOXC-AS2/miR-876-5p/HKDC1 signal transduction axis regulated tumor microenvironment (TME) formation by enhancing glycolysis, promoting a metabolic advantage in lactate-rich environments to further accelerate EC progression.	[158]
		Lung cancer	LINC00969 interacted with EZH2 and METTL3, epigenetically repressing NLRP3 expression to suppress the activation of the NLRP3/caspase-1/GSDMD-related classical pyroptosis signalling pathways in lung cancer.	[159]
		Pancreatic adenocarcinoma (PAAD)	LINC01133 functioned as a competing endogenous RNA to sequester miR-30b-5p from sponging SIRT1 mRNA to inhibit PAAD pyroptosis.	[160]
Necroptosis	Histone modification	Breast cancer	The G9a-mediated silencing of pro-necroptotic proteins was a critical step in tumor recurrence.	[161]
			Histone deacetylase (HDAC) inhibitor hindered the progression of breast cancer by significantly up-regulating phospho-RIP3 and MLKL levels and inducing necroptosis.	[162]
		Glioblastoma	HDAC inhibitor induced glioma stem cell death via both apoptosis and necroptosis pathway.	[163]
		Acute myeloid leukemia (AML)	R-2HG induced RIPK1-dependent necroptosis via KDM2B inhibition in AML cells.	[164]
	DNA methylation	CRC	The key player of DNA methylation, UHRF1, regulated necroptosis by methylating the promoter of RIP3.	[165]
		Nasopharyngeal carcinoma	EBV infection inhibited necroptosis signaling by the methylation of the RIP3 promoter.	[166]
		Mesothelioma	RIPK3 functioned as a tumor suppressor in mesothelioma, and DNA methylation-mediated RIPK3 silence impeded necroptosis and contributed to cancer progression.	[167]
		NSCLC	The necroptosis pathway was suppressed in lung cancer through RIP3 promoter methylation, and reactivating this pathway should be exploited for improving lung cancer chemotherapy.	[168]
	Noncoding RNA	RCC	Necroptosis-related lncRNAs were selected by WGCNA; among them, RP11-133F8.2 and RP11-283G6.4 could serve as independent prognostic factors for clear cell renal cell carcinoma.	[169]
			miR-381-3p acted as an oncogenic miRNA through inhibiting the activation of RIPK3 and MLKL to block necroptosis.	[170]
		Glioma	Necroptosis-related lncRNAs were screened based on the risk score and could be helpful to predict the prognosis of glioma patients.	[171]
Ferroptosis	Histone modification	HCC	Upon ferroptosis induction, RB1-inducible coiled-coil 1 (RB1CC1) recruited the elongator acetyltransferase complex subunit 3 (ELP3) to strengthen H4K12Ac histone modifications within enhancers linked to ferroptosis and stimulated the transcription of ferroptosis-associated genes.	[172]
		Pancreatic ductal adenocarcinoma (PDAC)	EP300 acetyltransferase promoted ferroptosis in human PDAC cells via the acetylation of the heat shock protein family A member 5 (HSPA5); acetylated HSPA5 loses its ability to inhibit lipid peroxidation and subsequent ferroptotic cell death.	[173]
		AML	PRMT1 knockout up-regulated acyl-CoA synthetase long-chain family member 1 (ACSL1), which acts as a ferroptosis promoter by increasing lipid peroxidation.	[174]
		MM	Multiple myeloma cells were sensitive to ferroptosis induction and epigenetic reprogramming by RSL3, and the altered expression of histone modifications associated with DNA repair and cellular senescence.	[175]
			Methyltransferase G9a inhibitor (DCG066) inhibited the proliferation and induced ferroptosis in MM cells via the Nrf2/HO-1 pathway.	[176]
		NSCLC	LSD1 inhibition down-regulated the expression of ATF4 through H3K9me2, which sequentially inhibits the expression of the cystine-glutamate antiporter (xCT) and decreases glutathione (GSH) production.	[177]
			SETD1A amplified WTAP expression through WTAPP1 up-regulation by mediating H3K4me3 modification in the WTAPP1 promoter region, thus promoting NSCLC cell proliferation and migration and inhibiting ferroptosis.	[178]
		CRC	Lysine acetyltransferase 2 A (KAT2A) modulated the histone acetylation of GPX4 to regulate the proliferation, metastasis, and ferroptosis of CRC cells.	[179]
		LUAD	Multiple histone modifications had the coregulatory mechanisms of key ferroptosis-related genes in LUAD.	[180]
		Cervical cancer (CC)	Hypoxia-like conditions enhanced the SUMOylation of KDM4A at the K471 locus specifically, repressed H3K9me3 levels, and up-regulated SLC7A11/GPX4 to enhance CC cell ferroptosis resistance.	[181]
	DNA methylation	GC	DNA methylation in the encoding gene of ELOVL5 and FADS1 rendered cells resistant to ferroptosis.	[182]
		Gall bladder cancer (GBC)	The down-regulation of RUNX3 was mediated by DNA methylation, which promoted the pathogenesis of gall bladder cancer through attenuating SLC7A11-mediated ferroptosis.	[183]
		HNSCC	A novel ferroptosis-related 16-DNA methylation signature that could be applied as an alternative tool to predict prognosis outcome in patients with HNSCC.	[184]
		Glioma	The methylation of LINC02587 could inhibit cellular proliferative, migrative, and invasive properties and induce ferroptosis within gliomas through the CoQ-FSP1 pathway.	[185]
			The DNA methylation level of lncRNA SNAI3-AS1 promoter reduced its expression and impeded the anti-tumor activity of erastin through ferroptosis.	[186]
		ALL	In ALL samples, the promoter of the gene coding for FSP1 was hypermethylated, silencing the expression of FSP1 and creating a selective dependency on GSH-centered anti-ferroptosis defenses.	[187]
	Noncoding RNA	EC	LncRNA FAM83H-AS1 inhibited ferroptosis in EC by recruiting DNMT1 to increase CDO1 promoter methylation level and inhibit its expression.	[188]
		Breast cancer	The LINC00665-miR-410-3p axis was identified as the most potential upstream ncRNA-related pathway of ferroptosis-related gene EMC2 in breast cancer.	[189]
		HCC	The CircIDE/miR-19b-3p/RBMS1 axis influenced HCC cell growth by regulating the expression of glutathione peroxidase 4 (GPX4) and ferroptosis.	[190]
		NSCLC	MiR-4443 regulated the expression of FSP1 in an m6A-dependent manner.	[191]
Cuproptosis	DNA methylation	Glioma	Cuproptosis-related gene-located DNA-methylation sites linked to patient prognosis/immune microenvironment were established.	[192]
		Cutaneous melanoma		[193]
		HCC		[194]
	Noncoding RNA	Colon adenocarcinoma (COAD)	Cuproptosis-related lncRNA signature was screened to predict patient prognosis and immune landscape in cancer.	[195]
		HCC		[196]
		Osteosarcoma		[197]
		Bladder cancer (BLCA)		[198]
		Pancreatic cancer (PC)		[199]
		HNSCC		[200]
		Uterine corpus endometrial carcinoma (UCEC)		[201]

**Table 3:** Drugs/inhibitors targeting cell death for cancer therapy.

Types	Drug/Small Compound	Target	Indications
Apoptosis	Venetoclax	BCL-2	Chronic myeloid leukaemia (CML), Chronic lymphocytic leukemia (CLL), AML, ALL
	Navitoclax		CLL
	ABT-737		AML, Pca
	APG-1252		NSCLC, GC, HCC
	Lisaftoclax (APG-2575)		AML, WM, MM, CLL/SLL, Follicular lymphoma (FL)
	BCL-201		FL, Mantle cell lymphoma (MCL)
	AZD4320		Lymphoma, MM
	Sonrotoclax (BGB-11417)		CLL/SLL, AML, Non-Hodgkin’s Lymphoma, Multiple myeloma
	AMG176	MCL1	AML, MM, CLL
	MIK665		HCC, MM, Lymphoma
	Xevinapant	IAP	Squamous cell carcinoma of the head and neck (SCCHN), PDAC, CRC
	AT-406		CC, CRC
	Tollinapant		Advanced malignant solid tumor, T-cell lymphoma
	APG-1387		Pca, Hepatitis B
	LCL161		HNSCC, MM, ALL
	APG-115	MDM2-p53	AML, GC
	Idasanutlin		AML, Neuroblastoma
	Nelfinavir	Protein kinase B (AKT) pathway	CC
	Mapatumumab	Apoptosis-inducing ligand receptor-1 (TRAIL-R1)	CC
Autophagy	Chloroquine (CQ)	Lysosome	CRC, Breast cancer
	HCQ		Breast cancer
	SBI-0206965	AMPK	LUAD, Glioblastoma
	3MA	PI3K	GC, Melanoma, Glioma
Pyroptosis	Disulfiram	GSDMD	NSCLC, Liver cancer, Breast cancer, Pca, PC
	Gambogic Acid	CNPY3	Pca
	Metformin	GSDME	Breast cancer, Colon cancer
	Docosahexaenoic acid	Caspase-1,GSDMD	Triple-negative breast cancer
	Simvastatin	Caspase-1	NSCLC
	Mirdametinib, Vemurafenib	MEK/ERK1/2	Melanoma
	Paclitaxel	Caspase-3/GSDME	Lung cancer
	Doxorubicin	Anti-tumor CD8^+^ T cells and NK cells	HNSCC
	Tetraarsenic hexoxide	Caspase-3/GSDME	Triple-negative breast cancer
	Antibody Targeting Gasdermin-B	GSDMB	Breast cancer
	Dimethyl fumarate (DMF)	GSDMD	Breast cancer
Necroptosis	Necrostatin-1	RIPK1	CRC
	FTY720	I2PP2A/SET	Lung cancer
	Chloroquine (CQ)	RIPK3	CRC
	Emodin	TNF/RIPK1/RIPK3	Glioma
	Ophiopogonin D’	RIPK1	Pca
	Tanshinol A	MLKL	Lung cancer
	CBL0137	ZBP1	Breast cancer, CRC, Melanoma
Ferroptosis	Erastin	VDAC2/VDCA3	Breast cancer, Melanoma, CC
	RSL3	GPX4	CRC
	Deferoxamine	Fenton reaction	Breast cancer, HCC
	β-mercaptoethanol	System x_c_^-^	NSCLC
	Ciclopirox	Intracellular iron chelator	GC
	Sorafenib	System x_c_^-^	HCC
	Cisplatin	GSH levels, GPXs	CRC
	Apatinib	GSH levels, GPX4	GC
	AS-252424 (AS)	ACSL4	Kidney ischemia/reperfusion injury, Acute liver injury (ALI)
	BRD4770	System Xc^-^-GPX4, FSP1-CoQ10, GCH1-BH4 pathways	Aortic dissection (AD)
	Sulfasalazine	System x_c_^-^	Rheumatoid arthritis, Brain cancer
